# Determinants of Microbial Load in Infected Diabetic Foot Ulcers: A Pilot Study

**DOI:** 10.1155/2013/858206

**Published:** 2013-06-26

**Authors:** M. Demetriou, N. Papanas, M. Panopoulou, K. Papatheodorou, E. Maltezos

**Affiliations:** ^1^Outpatient Clinic of the Diabetic Foot, Second Department of Internal Medicine, Democritus University of Thrace, University Hospital of Alexandroupolis, Dragana, 68100 Alexandroupolis, Greece; ^2^Microbiology Laboratory, Democritus University of Thrace, University Hospital of Alexandroupolis, Dragana, 68100 Alexandroupolis, Greece

## Abstract

We examined the determinants of microbial load in infected diabetic foot ulcers in 62 patients (38 men and 24 women, mean age: 65.63 ± 12.71 years) with clinically infected diabetic foot ulcers. Tissue cultures were taken from ulcers by 4 mm punches. Ulcer grade (University of Texas classification), neuropathy disability score (NDS), neuropathy symptom score (NSS), ankle-brachial index (ABI), perfusion, extent, depth, infection, and sensation (PEDIS) grade of diabetic foot infection, and laboratory parameters were evaluated in all patients. Total microbial load was positively correlated with the number of isolates on tissue cultures (*r*_*s*_ = 0.544, *P* < 0.001), white blood cell count (WBC) (*r*_*s*_ = 0.273, *P* = 0.032), and platelet count (PLT) (*r*_*s*_ = 0.306, *P* = 0.015). It also exhibited a borderline insignificant positive correlation with PEDIS infection grade (*r*_*s*_ = 0.246, *P* = 0.053). In stepwise linear regression analysis, the number of isolates on tissue cultures and WBC were identified as the only two significant parameters accounting for 38% of the variation in the log of total microbial load (adjusted *R* ^2^ = 0.380, *P* < 0.001). In conclusion, patients with infected diabetic foot ulcer exhibit a positive correlation of total microbial load with the number of isolates on tissue cultures, WBC and PLT.

## 1. Introduction

In both epidemiological surveys and everyday clinical practice, the diabetic foot remains a major cause of patient morbidity [1–3] and nontraumatic lower extremity amputations [4–6]. Ischaemia, neuropathy, and infection are the three cardinal aetiological factors predisposing to diabetic foot ulcers [3, 7]. Some progress has been accomplished in the management of these conditions [7], including revascularisation [8] and improved pharmacology for peripheral arterial disease [9], neuroprotective agents [10, 11], new antibiotics [12, 13], growth factors [14, 15], and adjunctive treatment modalities [16–18], but there is a considerably long way to go to improve outcomes [2, 4, 7].

 In particular, diabetic foot infections may be extremely challenging to cure [3, 7, 19]. Some of the therapeutic difficulty arises from late diagnosis (due to blunted clinical signs [3, 7, 13]), presence of ischaemia [7, 20], difficult-to-treat Methicillin-resistant* Staphylococcus aureus* (MRSA) [21, 22] or other multidrug-resistant pathogens, and spread of infection to the bones, leading to osteomyelitis [23–25]. Characteristics of foot ulcers (chronicity, extension, and depth), prior antibiotic use, and presence of peripheral arterial disease, generally, have a considerable impact on bacterial pathogens in infected foot ulcers [7, 25–28], but there is no reliable way of predicting types of pathogens and microbial load [7, 13]. This is important because a high number of foot ulcers are nowadays already infected at initial presentation [29]. Therefore, the aim of the present study is to examine the determinants of microbial load in infected diabetic foot ulcers.

## 2. Patients and Methods

 This study included 62 patients with clinically infected diabetic foot ulcers presenting to the Outpatient Clinic of the Diabetic Foot of the Second Department of Internal Medicine at Democritus University of Thrace, Greece. Patient characteristics are presented in Table 1. The study was approved by the institutional ethics committee and all patients gave their informed consent.

Foot ulcers were defined as skin defects located beneath the malleoli and extending through all skin layers [30, 31]. Infection of foot ulcers was based on clinical presentation, requiring ≥2 of the following criteria: local swelling or induration; erythema greater than 0.5 cm in any direction around the ulcer; local tenderness or pain; local increase of temperature; purulent discharge [13]. Ulcer grade was based on the University of Texas (UT) classification system [32], and clinical severity of infection was quantified according to the PEDIS system proposed by the International Working Group on the Diabetic Foot [31]. Ulcer duration was measured in months, as based on medical history of tissue breakdown. Diagnosis of osteomyelitis was based on positive probe-to-bone test and/or positive magnetic resonance imaging [33]. Patients with osteomyelitis were excluded. 

 Moreover, patients were examined for diabetic polyneuropathy by the neuropathy disability score (NDS), a standardised clinical examination system incorporating loss of ankle reflexes and sensory deficits in the feet [34]. Peripheral arterial supply was evaluated by the ankle-brachial index (ABI) measured by a Doppler apparatus [35, 36]. Neuropathic symptoms were assessed by the neuropathy symptom score (NSS) [34]. Glycated haemoglobin (HbA_1c_), c-reactive protein (CRP), full blood count, and biochemical parameters were measured in blood samples. 

 Patients had not been treated with antibiotics for 1 week prior to examination. Following appropriate debridement, deep-tissue cultures were taken from ulcers by 4 mm biopsy punches (Kai Europe GmbH, Solingen, Germany), as previously described [28]. Specimens were placed in sterile transport containers, which were delivered to the Microbiology Laboratory within 20 minutes. 

 Quantitative tissue cultures were performed using standardised procedures [37], as described in [28]. Tissue specimens were weighed, homogenised, and diluted with 5 mL of Thioglycolate broth. Serial 10-fold dilutions to 10^4^ were made with 0.85% NaCl, and 0.1 mL of each dilution was plated onto the appropriate media. Samples for aerobic cultures were inoculated into Columbia sheep blood agar and MacConkey agar plates and were then incubated at 35°C for 24–48 hours. Samples for anaerobic cultures were inoculated into Brucella agar with 5% sheep blood supplemented with vitamin K and haemin and were then incubated at 35°C for 48–72 hours in anaerobic jars (Gas Pak EZ Gas Generating Container System, Becton Dickinson, Sparks, MD, USA). Identification of species was based on the automated system Vitek 2 and the Api 20A (BioMerieux, Marcy l' Etoile, France) [28]. Total microbial load was expressed as number of colony-forming units (CFUs) per g of tissue.

 Statistical analysis was performed with Statistical Package for Social Sciences (SPSS, Chicago, IL, USA) version 19. Normality of distribution was evaluated by Kolmogorov-Smirnov test. Normally distributed variables were expressed as mean ± Standard Deviation and variables without normal distribution as median and range. Correlations of isolate numbers and total microbial load were examined by Spearman's rank coefficient. We also performed stepwise linear regression analysis using log of total microbial load as independent variable. Significance was defined at the 5% level (two-tailed  *P* < 0.05).

## 3. Results

Correlations of total microbial load are presented in Table 2. Total microbial load was positively correlated with the number of isolates on tissue cultures (*r*_*s*_ = 0.544,  *P* < 0.001), white blood cell count (WBC) (*r*_*s*_ = 0.273,  *P* = 0.032), and platelet count (PLT) (*r*_*s*_ = 0.306,  *P* = 0.015). It also exhibited a borderline insignificant positive correlation with PEDIS grade of diabetic foot infection (*r*_*s*_ = 0.246,  *P* = 0.053). Moreover, the number of isolates on tissue cultures exhibited a positive correlation with platelet count (*r*_*s*_ = 0.339,  *P* = 0.007).

 In stepwise linear regression analysis, log of total microbial load was used as independent variable, while dependent variables included number of isolates on tissue cultures, age, diabetes duration, ulcer duration, PEDIS grade of diabetic foot infection, ulcer grade, WBC, PLT, HbA_1c_, ABI, NDS, and NSS. The number of isolates on tissue cultures and WBC were identified as the only two significant parameters accounting for 38% of the variation in the log of total microbial load (adjusted  *R* ^2^ = 0.380,  *F* = 19.712,  *P* < 0.001).

## 4. Discussion

 The present study found a positive correlation between total microbial load and the number of isolates on tissue cultures. Indeed, the latter was one of the 2 parameters significantly influencing the variation of the former. This is not surprising, given that total microbial load was calculated by adding CFUs per g tissue of each isolate. In practice, our finding shows a high microbial load to be encountered in polymicrobial infections. Consequently, the clinician should be alerted that a high microbial load calls for aggressive antibiotic management with use of agents targeting multiple pathogens.

 Moreover, total microbial load exhibited a positive correlation with some markers of inflammation, that is, WBC and PLT, though not with CRP. WBC was the other parameter significantly contributing to the variation of total microbial load in stepwise linear regression analysis. Inflammatory markers have been used in the study of foot infections [38–41]. The commonest applications include diagnosis of infection [39, 40], distinction between soft-tissue infection and osteomyelitis [38, 41], as well as differential diagnosis of infection from Charcot osteoarthropathy, in which serum markers are, generally, normal [42]. This study adds the association of WBC and PLT with high microbial load in patients with clinically infected diabetic foot ulcers. 

 We also found a borderline insignificant correlation of total microbial load with clinical severity of infection, as expressed by the PEDIS grade of diabetic foot infection [31]. This finding should be interpreted in the light of available evidence that type and number of microorganisms cannot be reliably predicted on the basis of clinical manifestation [1, 7, 12, 13]. Essentially, a number of factors may influence the clinical manifestation of infection, notably ischaemia and neuropathy, both of which may blunt inflammatory response [3, 7, 43]. An alternative explanation for the absence of significant correlation is the homogeneity of our study population, inasmuch as patients had infection grade PEDIS 2 or 3 only. 

 Conversely, total microbial load exhibited no association with ulcer duration. Generally, long-standing ulcers are predisposed to colonisation and infection [1, 7, 12, 13]. However, this propensity does not equate to development of high microbial load, as shown by our findings. Indeed, infection has now been documented to be very common even at initial presentation of diabetic foot ulcers [29]. 

 Of note, there was no association between total microbial load and ulcer stage. This is most likely due to the fact that all patients had UT stage 1 or 2 ulcers and not more severe lesions, so that such relationship could not be documented. We also found no association of total microbial load with age and diabetes duration. This agrees with current knowledge that the aforementioned factors do not relate to the severity of foot infections [3, 7].

 Interestingly, total microbial load did not correlate with ABI. We have previously found no difference in the number of isolates on tissue cultures, in the frequency of high microbial load, and in the number of CFUs/g tissue between patients with neuropathic and those with neuroischaemic infected foot ulcer [28]. Based on the new and on the prior data, it is plausible that the adequacy of arterial blood flow itself is not of paramount importance in determining microbial load among patients with infected diabetic foot ulcer. Instead, the impact of ischaemia is crucial on treatment outcomes [7, 44], which should not be overlooked in clinical practice [2]. 

 Similarly, total microbial load did not correlate with clinical severity of neuropathy (NDS) and of neuropathic symptoms (NSS). Arguably, this novel finding may be seen as increasing our knowledge on the pathogenic role of neuropathy. While patients with more severe neuropathy are at increased risk of foot ulceration [1, 2, 30, 45], and while ulceration, in turn, increases the risk of superimposed infection, severity of neuropathy per se appears not to affect the total microbial load. This does not negate the pivotal role of neuropathy in the development of foot ulceration [1–3, 45–47] but shows that other factors determine microbial load in the case of infection complicating ulceration.

 The strengths of this study are the use of quantitative tissue cultures and the homogeneous study population. Indeed, microbial load was quantified on deep-tissue specimens and not on superficial swabs, which may, generally, be criticised for harbouring contaminating skin flora as well [12, 13]. Of particular importance, we excluded patients with osteomyelitis, in whom the microbial load of infected bones and not of soft tissues would be relevant [13, 23, 33]. The limitations include the relatively small patient series and the underrepresentation of type 1 diabetes, given that the vast majority of patients had type 2 diabetes. Of additional note, all patients presented with clinically infected foot ulcers. Hence, our results cannot be readily extrapolated to subjects with uncomplicated foot lesions. 

 The clinical implications of our findings may be summarised as follows. In patients with clinically infected diabetic foot ulcers, microbial load is associated with increased number of isolates on tissue cultures, as well as elevated WBC, and PLT counts. It is conceivable that this information may prove useful for the choice of initial empirical antibiotic regimen, inasmuch as patients with elevated WBC and/or PLT may be taken to require more aggressive antibiotic regimen with broad coverage for a polymicrobial infection. However, more experience with this interpretation is desirable. 

 In conclusion, our results indicate that patients with infected diabetic foot ulcers exhibit a positive correlation of total microbial load with number of isolates on tissue cultures, WBC and PLT. Conversely, no such association is seen with severity of ischaemia and peripheral neuropathy. These findings should be seen in the context of the clinician's attempt to estimate severity of infection and choose the initial antibiotic regimen. Findings reported herein might prove useful in this endeavour, but further confirmation is awaited.

## Figures and Tables

**Table 1:** Patient characteristics.

Parameter	Value
Age (years, mean ± SD)	65.63 ± 12.71
Sex (males/females)	38/24
Diabetes type (2/1)	59/3
Diabetes duration (years, mean ± SD)	16.31 ± 8.07
Ulcer duration (median, IQR)*	2 (1.0–4.25)
ABI (mean ± SD)	0.94 ± 0.34
NDS (mean ± SD)	7.02 ± 2.33
NSS (median, IQR)	2 (0–4)
PEDIS infection grade (median, IQR)	2 (2-3)
Total microbial load (CFU_S_, median, IQR)	275000 (0.0–24900000)
Number of isolates on tissue culture (median, IQR)	1 (0–2)
WBC (mean ± SD)	8142.58 ± 2174.97
PLT (mean ± SD)	271685.48 ± 81581.41
Ht% (mean ± SD)	36.77 ± 4.56
CRP (mg/dL, median, IQR)	1.2 (0.72–2.93)
Urea (mg/dL, median, IQR)	41.50 (34.00–56.25)
Creatinine (mg/dL, median, IQR)	1.00 (0.80–1.20)
AST (U/L, mean ± SD)	22.85 ± 9.84
ALT (U/L, mean ± SD)	24.39 ± 10.79
HbA_1c_ (%, mean ± SD)	8.29 ± 1.50
LDL (mg/dl, mean ± SD)	114.53 ± 38.65
HDL (mg/dl, mean ± SD)	44.07 ± 10.82
TC (mg/dl, mean ± SD)	189.32 ± 44.83
TGs (mg/dl, mean ± SD)	159.61 ± 50.26
SUA (mg/dl, mean ± SD)	5.24 ± 1.62
CPK (U/L, mean ± SD)	126.03 ± 78.18
FPG (mg/dl, mean ± SD)	171.18 ± 56.65

*Duration of ulcer in months, as based on medical history.

ABI: ankle-brachial index; ALT: alanine aminotransferase; AST: aspartate aminotransferase; CFUs: colony-forming units; CPK: creatine phosphokinase; CRP: c-reactive protein; FPG: fasting plasma glucose; HDL: high-density lipoprotein cholesterol; Ht%: haematocrit; IQR: interquartile range; LDL: low-density lipoprotein cholesterol; NDS: neuropathy disability score; NSS: neuropathy symptom score; PEDIS: grade of diabetic foot infection, according to the International Working Group on the Diabetic Foot; PLT: platelet count; SD: standard deviation; SUA: serum uric acid; TC: total cholesterol; TGs: triglycerides; WBC: white blood cell count.

**Table 2:** Correlations of total microbial load.

Correlations of total microbial load	*P* value
Number of isolates on tissue cultures	*r*_*s*_ = 0.544, *P* < 0.001
WBC	*r*_*s*_ = 0.273, *P* = 0.032
PLT	*r*_*s*_ = 0.306, *P* = 0.015
PEDIS infection grade	*r*_*s*_ = 0.246, *P* = 0.053
Age	*r*_*s*_ = − 0.147, *P* = 0.253
Diabetes duration	*r*_*s*_ = − 0.20, *P* = 0.880
Ulcer duration*	*r*_*s*_ = 0.048, *P* = 0.712
UT ulcer stage	*r*_*s*_ = 0.202, *P* = 0.116
NDS	*r*_*s*_ = − 0.119, *P* = 0.359
ABI	*r*_*s*_ = − 0.032, *P* = 0.806
NSS	*r*_*s*_ = − 0.101, *P* = 0.436
Ht%	*r*_*s*_ = 0.051, *P* = 0.694
CRP	*r*_*s*_ = 0.140, *P* = 0.278
Urea	*r*_*s*_ = − 0.005, *P* = 0.969
Creatinine	*r*_*s*_ = 0.048, *P* = 0.710
AST	*r*_*s*_ = 0.000, *P* = 1.000
ALT	*r*_*s*_ = − 0.068, *P* = 0.599
HbA_1c_	*r*_*s*_ = 0.228, *P* = 0.075
LDL	*r*_*s*_ = 0.113, *P* = 0.382
HDL	*r*_*s*_ = 0.051, *P* = 0.692
TC	*r*_*s*_ = 0.130, *P* = 0.315
TGs	*r*_*s*_ = 0.104, *P* = 0.420
SUA	*r*_*s*_ = 0.096, *P* = 0.458
CPK	*r*_*s*_ = − 0.197, *P* = 0.125
FPG	*r*_*s*_ = 0.015, *P* = 0.908

*Duration of ulcer in months, as based on medical history.

ABI: ankle-brachial index; ALT: alanine aminotransferase; AST: aspartate aminotransferase; CPK: creatine phosphokinase; CRP: c-reactive protein; FPG: fasting plasma glucose; HDL: high-density lipoprotein cholesterol; Ht%: haematocrit; LDL: low-density lipoprotein cholesterol; NDS: neuropathy disability score; NSS: neuropathy symptom score; PEDIS: grade of diabetic foot infection, according to the International Working Group on the Diabetic Foot; PLT: platelet count; SUA: serum uric acid; TC: total cholesterol; TGs: triglycerides; UT: University of Texas; WBC: white blood cell count.

## References

[1] Boulton AJM (2008). The diabetic foot: grand overview, epidemiology and pathogenesis. *Diabetes/Metabolism Research and Reviews*.

[2] Papanas N, Maltezos E, Edmonds M (2011). Salvation of the diabetic foot: still a quest for the holy grail?. *Vasa*.

[3] Papanas N, Maltezos E (2009). The diabetic foot: a global threat and a huge challenge for Greece. *Hippokratia*.

[4] Papanas N, Lazarides MK (2011). Diabetic foot amputations in Greece: where do we go from here?. *International Journal of Lower Extremity Wounds*.

[5] Skoutas D, Papanas N, Georgiadis GS (2009). Risk factors for ipsilateral reamputation in patients with diabetic foot lesions. *International Journal of Lower Extremity Wounds*.

[6] Tentolouris N, Al-Sabbagh S, Walker MG, Boulton AJM, Jude EB (2004). Mortality in diabetic and nondiabetic patients after amputations performed from 1990 to 1995: a 5-year follow-up study. *Diabetes Care*.

[7] Edmonds M Modern treatment of infection and ischaemia to reduce major amputation in the diabetic foot.

[8] Georgakarakos E, Papanas N, Papadaki E, Georgiadis GS, Maltezos E, Lazarides MK Endovascular treatment of critical ischemia in the diabetic foot: new thresholds, new anatomies.

[9] Paraskevas KI, Athyros VG, Briana DD, Kakafika AI, Karagiannis A, Mikhailidis DP (2007). Statins exert multiple beneficial effects on patients undergoing percutaneous revascularization procedures. *Current Drug Targets*.

[10] Papanas N, Maltezos E (2012). *α*-lipoic acid, diabetic neuropathy, and Nathan’s prophecy. *Angiology*.

[11] Papanas N, Maltezos E (2011). Cilostazol in diabetic neuropathy: premature farewell or new beginning?. *Angiology*.

[12] Lipsky BA, Peters EJG, Berendt AR (2012). Specific guidelines for the treatment of diabetic foot infections 2011. *Diabetes/Metabolism Research and Reviews*.

[13] Lipsky BA, Peters EJG, Senneville E (2012). Expert opinion on the management of infections in the diabetic foot. *Diabetes/Metabolism Research and Reviews*.

[14] Papanas N, Maltezos E (2007). Growth factors in the treatment of diabetic foot ulcers: new technologies, any promises?. *International Journal of Lower Extremity Wounds*.

[15] Tiaka EK, Papanas N, Manolakis AC, Georgiadis GS (2012). Epidermal growth factor in the treatment of diabetic foot ulcers: an update. *Perspectives in Vascular Surgery and Endovascular Therapy*.

[16] Tiaka EK, Papanas N, Manolakis AC, Maltezos E (2012). The role of hyperbaric oxygen in the treatment of diabetic foot ulcers. *Angiology*.

[17] Antonoglou C, Papanas N, Maltezos E Lipid-lowering therapy in the diabetic foot: seeing the whole iceberg and not just the tip.

[18] Papanas N, Maltezos E (2011). Polyherbal formulation as a therapeutic option to improve wound healing in the diabetic foot. *Indian Journal of Medical Research*.

[19] Aragón-Sánchez J, Lázaro-Martínez JL, Pulido-Duque J, Maynar M (2012). From the diabetic foot ulcer and beyond: how do foot infections spread in patients with diabetes?. *Diabetic Foot and Ankle*.

[20] Aragón-Sánchez J, Lázaro-Martínez JL, Hernández-Herrero C (2011). Surgical treatment of limb- and life-threatening infections in the feet of patients with diabetes and at least one palpable pedal pulse: successes and lessons learnt. *International Journal of Lower Extremity Wounds*.

[21] Tentolouris N, Petrikkos G, Vallianou N (2006). Prevalence of methicillin-resistant Staphylococcus aureus in infected and uninfected diabetic foot ulcers. *Clinical Microbiology and Infection*.

[22] Eleftheriadou I, Tentolouris N, Argiana V, Jude E, Boulton AJ (2010). Methicillin-resistant staphylococcus aureus in diabetic foot infections. *Drugs*.

[23] Aragón-Sánchez J, Lipsky BA, Lázaro-Martínez JL (2013). Gram-negative diabetic foot osteomyelitis: risk factors and clinical presentation. *The International Journal of Lower Extremity Wounds*.

[24] Cecilia-Matilla A, Lázaro-Martínez JL, Aragón-Sánchez J (2013). Histopathologic characteristics of bone infection complicating foot ulcers in diabetic patients. *Journal of the American Podiatric Medical Association*.

[25] Wheat LJ, Allen SD, Henry M (1986). Diabetic foot infections. Bacteriologic analysis. *Archives of Internal Medicine*.

[26] Sharp CS, Bessman AN, Wagner FW, Garland D (1978). Microbiology of deep tissue in diabetic gangrene. *Diabetes Care*.

[27] Sapico FL, Canawati HN, Witte JL (1980). Quantitative aerobic and anaerobic bacteriology of infected diabetic feet. *Journal of Clinical Microbiology*.

[28] Demetriou M, Papanas N, Panopoulou M, Papatheodorou K, Bounovas A, Maltezos E (2013). Tissue and swab culture in diabetic foot infections: neuropathic versus neuroschaemic ulcers. *The International Journal of Lower Extremity Wounds*.

[29] Prompers L, Huijberts M, Apelqvist J (2007). High prevalence of ischaemia, infection and serious comorbidity in patients with diabetic foot disease in Europe. Baseline results from the Eurodiale study. *Diabetologia*.

[30] Edmonds ME, Foster AVM, Sanders LJ, Edmonds ME, Foster AVM, Sanders LJ (2004). Stage 3: the ulcerated foot. *A Practical Manual of Diabetic Footcare*.

[31] Schaper NC (2004). Diabetic foot ulcer classification system for research purposes: a progress report on criteria for including patients in research studies. *Diabetes/Metabolism Research and Reviews*.

[32] Lavery LA, Armstrong DG, Harkless LB (1996). Classification of diabetic foot wounds. *Journal of Foot and Ankle Surgery*.

[33] Byren I, Peters EJG, Hoey C, Berendt A, Lipsky BA (2009). Pharmacotherapy of diabetic foot osteomyelitis. *Expert Opinion on Pharmacotherapy*.

[34] Young MJ, Boulton AJM, Macleod AF, Williams DRR, Sonksen PH (1993). A multicentre study of the prevalence of diabetic peripheral neuropathy in the United Kingdom hospital clinic population. *Diabetologia*.

[35] Papanas N, Symeonidis G, Mavridis G (2007). Ankle-brachial index: a surrogate marker of microvascular complications in type 2 diabetes mellitus?. *International Angiology*.

[36] Paraskevas KI, Kotsikoris I, Koupidis SA, Giannoukas AD, Mikhailidis DP (2010). Editorial: Ankle-brachial index: a marker of both peripheral arterial disease and systemic atherosclerosis as well as a predictor of vascular events. *Angiology*.

[37] York MK, Isenberg HD (2004). Aerobic Bacteriology. Quantitative cultures of wound tissues. *Clinical Microbiology Procedures Handbook*.

[38] Rabjohn L, Roberts K, Troiano M, Schoenhaus H (2007). Diagnostic and prognostic value of erythrocyte sedimentation rate in contiguous osteomyelitis of the foot and ankle. *Journal of Foot and Ankle Surgery*.

[39] Pepys MB, Hirschfield GM (2003). C-reactive protein: a critical update. *Journal of Clinical Investigation*.

[40] Dinh T, Snyder G, Veves A (2010). Current techniques to detect foot infection in the diabetic patient. *International Journal of Lower Extremity Wounds*.

[41] Michail M, Jude E, Liaskos C (2013). The performance of serum inflammatory markers for the diagnosis and follow-up of patients with osteomyelitis. *The International Journal of Lower Extremity Wounds*.

[42] Gouveri E, Papanas N (2011). Charcot osteoarthropathy in diabetes: a brief review with an emphasis on clinical practice. *World Journal of Diabetes*.

[43] Edmonds ME, Foster AVM, Sanders LJ, Edmonds ME, Foster AVM, Sanders LJ (2004). Stage 4: the infected foot. *A Practical Manual of Diabetic Footcare*.

[44] Aragón-Sánchez J, Lázaro-Martínez JL, Hernández-Herrero C (2011). Surgical treatment of limb- and life-threatening infections in the feet of patients with diabetes and at least one palpable pedal pulse: successes and lessons learnt. *International Journal of Lower Extremity Wounds*.

[45] Abbott CA, Carrington AL, Ashe H (2002). The North-West Diabetes Foot Care Study: incidence of, and risk factors for, new diabetic foot ulceration in a community-based patient cohort. *Diabetic Medicine*.

[46] Papanas N, Vinik AI, Ziegler D (2011). Neuropathy in prediabetes: does the clock start ticking early?. *Nature Reviews Endocrinology*.

[47] Papanas N, Boulton AJ, Malik RA (2013). A simple new non-invasive sweat indicator test for the diagnosis of diabetic neuropathy. *Diabetic Medicine*.

